# The Efficacy of Shinbaro for the Preventive Treatment of Migraine: A Pilot Study

**DOI:** 10.1155/2019/2363420

**Published:** 2019-05-13

**Authors:** Yesol Jung, Bohee Won, Mijung Lee, Jinyoung Chung, Sung Ju Han, Manho Kim

**Affiliations:** ^1^Department of Neurology, Seoul National University Hospital, Seoul, Republic of Korea; ^2^Interdisciplinary Program in Neuroscience, Seoul National University, Seoul, Republic of Korea; ^3^Department of Veterinary Medicine, Kangwon National University, Chuncheon, Republic of Korea

## Abstract

**Objective:**

To investigate the therapeutic potential and efficacy of Shinbaro, an herbal medication for inflammatory diseases and bone disorders, as a preventive treatment of migraine.

**Methods:**

In this prospective, interventional, single-arm, pre-post study, 37 migraine patients took 600mg bid of Shinbaro for 12 weeks. At 4-week intervals, the migraine frequency and the rescue medications frequency were measured from each patient's headache diary. The modified Migraine Disability Assessment (MIDAS) questionnaires to assess migraine associated disabilities were also completed at each visit. The serum calcitonin gene-related peptide (CGRP) concentrations before and after 12 weeks of Shinbaro administration were compared.

**Results:**

The monthly migraine frequency was significantly reduced from 20.5 days at baseline to 16.4 days at week 12 (*P *=0.003), and 22% of the participants showed ≥ 50% reduction. The frequency reduction was observed by week 4 (*P* =0.035) and continuously occurred through week 8 (*P* =0.001) and week 12 (*P* =0.003). The rescue medications frequency also decreased significantly from 17.4 days at baseline to 13.2 days at week 12 (*P* =0.035). Lastly, the serum CGRP concentration dropped from 434.6 pg/mL at baseline to 371.4 pg/mL at week 12, which was statistically significant (*P *<0.001).

**Conclusions:**

Shinbaro demonstrated prophylactic effects in migraine patients, significantly reducing their mean migraine frequency, rescue medications frequency, and the serum CGRP concentration after 12 weeks of treatment. This study is registered in Clinical Research Information Service, Seoul National University Hospital Clinical Research Institute (IRB No. 1604-138-758).

## 1. Introduction

First-line acute migraine medications such as nonsteroidal anti-inflammatory drugs (NSAIDs) and oral triptans (i.e., 5-HT1B/1D receptor agonists) can abort migraine and its associated symptoms within 30 minutes of administration, if they are effective [1]. However, since therapeutic response to a single treatment is usually low, most patients are prescribed several classes of medications. The problem is that increased dependence on the acute treatments can complicate migraine over time, by increasing the risks of chronic migraine (CM), medication-overuse headache (MOH), and intractable or status migraines, especially for the episodic migraine (EM) patients [2–4]. Meanwhile, *β*-blockers, anticonvulsants, 5-HT2 antagonists, tricyclic antidepressants, and calcium antagonists are commonly prescribed as migraine prophylaxes that are daily administered to reduce the frequency and severity of migraine and enhance patients' therapeutic responses to the acute treatments [5, 6]. However, various side effects ranging from mild fatigues to more severe cases of cognitive impairments and even Parkinsonian symptoms have been commonly reported [7, 8]. Treatments that are effective yet safe are continue to be investigated.

The pathophysiology of migraine involves the activation of trigeminovascular pathway and neurogenic inflammation, which are inseparable processes that are largely mediated by a multifunctional neuropeptide called calcitonin gene-related peptide (CGRP) [9, 10]. Peripherally, CGRP triggers vasodilation of the sensory fibers and degranulation of the mast cells. In consequence, proinflammatory cytokines such as bradykinin, histamine, and tumor necrosis factor-*α* (TNF-*α*) are released from the brain and further stimulate the release of CGRP in a positive feedback loop [11–13]. This cascade of inflammatory reactions along the central and peripheral nervous systems sensitizes the meningeal nociceptors and arouses migraine associated symptoms and pains [11–13].

Shinbaro is an herbal medicine that has been approved for treating bone and joint disorders and several inflammatory conditions. Preclinical studies with osteoarthritis (OA) rats have demonstrated that Shinbaro ameliorates pain-related behaviors by increasing the pain thresholds, and also by inhibiting the CGRP expression through the suppression of proinflammatory cytokines [14–18]. In a series of clinical studies in OA patients, Shinbaro exhibited antinociceptive and anti-inflammatory functions that were noninferior to those of cyclooxygenase-2 (COX-2) inhibitors and NSAIDs [19, 20]. Because the migraine pathophysiology also involves neurogenic inflammation and sensitization of the nociceptive neurons, we proposed that the therapeutic effects of Shinbaro may be extended to the treatment of migraine. The efficacy of Shinbaro as a migraine prophylaxis was investigated for the first time. 

## 2. Methods

### 2.1. The Study Drug

Shinbaro® is a prescription drug that is manufactured and sold by GC Pharma (formerly called Green Cross Corporation, Yongin, Korea). It is composed of the following six oriental herbs that are commonly used in the traditional East Asian medicines to treat several bone disorders:* Ledebouriellae* Radix (*Fang Feng*),* Achyranthis *Radix (*Huai Niu Xi*),* Acanthopanacis *Cortex (*Wu Jia Pi*),* Cibotii *Rhizoma (*Gou Ji*),* Glycine *Semen (*Hei Dou*), and* Eucommiae *Cortex (*Du Zhong*) [19]. A number of in vitro and in vivo studies have revealed that these herbal components contribute to the anti-inflammatory or the analgesic properties of Shinbaro or both of them, although* Acanthopanacis *Cortex seemed to have specific antiarthritic effect in OA [16, 17, 19, 21–23]. A tablet of Shinbaro contains 300mg of a dried extract from the six herbs, and two tablets are orally administered twice a day. It was approved as a treatment for bone disorders and inflammatory conditions by the Korea Food and Drug Administration (FDA) in 2011.

### 2.2. Subject Eligibility

This study was conducted in migraine patients who had visited the neurology clinic at Seoul National University Hospital and referred to the study by the physician. Eligible participants were women and men of ages between 19 and 65, who were diagnosed with migraine according to the criteria of the International Classification of Headache Disorders-III guidelines, had ≥ 4 migraine days in a month for at least three months, and could read and complete the migraine questionnaires. Women of child-bearing ages agreed to use contraceptives and showed negative pregnancy test results. Individuals who fell into one or more of the following criteria were excluded from the study: (1) experiencing migraine attacks at baseline or during the treatment period due to a substance withdrawal, (2) first onset of migraine occurring later than the age of 50, (3) having progressive neurological diseases, peptic ulcer, cardiovascular diseases, or any other serious medical conditions that could pose safety issues or influence the study results, (4) having impaired hepatic or renal functions with the blood creatinine, AST, ALT, and total bilirubin levels being ≥ 1.5 times greater than the upper limit of the normal range, (5) being allergic to NSAIDs or to the study drug, (6) women being pregnant or lactating, (7) participating in any other clinical studies, (8) having taken one or more of the migraine prophylactic drugs such as propranolol, flunarizine, verapamil, tricyclic antidepressant, valproic acid, topiramate, and gabapentin, within four weeks prior to the study participation, (9) having a history of substance or alcohol abuse. 

### 2.3. Design and Protocol

This study was designed as a prospective, interventional, single-arm, pre-post study and received a written approval from the Institutional Review Boards (IRB) of Seoul National University Hospital (https://cris.snuh.org/ncris/). All subjects provided informed consent before participating in the study. Eligible participants self-administered the indicated daily dose or 600mg bid of Shinbaro for 12 weeks. The dose was not to be titrated, and any other migraine prophylaxes were prohibited during the study. NSAIDs and tryptamine-based medications to abort migraine attacks were permitted and recorded for the rescue medications frequency. Each participant returned every four weeks with a ± five-day visit window, submitted the headache diary which recorded the monthly migraine headache days, and completed the modified Migraine Disability Assessment Score (MIDAS) questionnaires, which measured the number of missed or affected days per month at home and at work due to migraine. In addition, patients reported any adverse event that had occurred since their last visits. Incentives were given to increase the participants' compliance. Blood samples were obtained at baseline and at week 12, and the serum CGRP levels were measured using the Human CGRP ELISA kit (Elabscience, Wuhan, China). Specifications for the ELISA kit can be obtained from the protocol provided by the manufacturer.

### 2.4. Study Objectives

The primary efficacy endpoint of this study was change in the participants' mean migraine frequency at week 12, compared with baseline. The secondary efficacy endpoints were the overall % reduction of the migraine frequency, % of the participants who showed ≥ 50% reduction, and changes in the mean rescue medications frequency and the mean MIDAS at week 12, compared with baseline. Furthermore, differences of the means at week 4 and week 8 vs. baseline were observed to examine the time-dependent therapeutic effects of Shinbaro. The exploratory endpoint was change in the mean serum CGRP concentration after 12 weeks of Shinbaro treatment.

### 2.5. Statistical Analysis

The sample size was determined by the central limit theorem to implement the following parametric tests. The paired sample t-test was used to compare the variable measures at week 12 vs. baseline, and the one-way repeated measures analysis of variance (ANOVA) was used to determine the time trend of Shinbaro efficacy, particularly for the variables that were measured at 4-week interval. The variables measures at week 4 and week 8 were each compared with the measures at baseline using the Fisher's least significant difference (LSD) t-tests without adjusting for multiple comparisons [24]. The efficacy analyses were conducted per protocol, including only the participants who had completed 12 weeks of Shinbaro administration without major protocol deviations, whereas the safety analysis included participants who had received at least one dose of the study medication. Statistical tests were two-sided, and* P* <0.05 was considered significant. All analyses were conducted on IBM SPSS version 23.0 (SPSS Inc., Chicago, IL, USA).

## 3. Results

**Figure 1:**
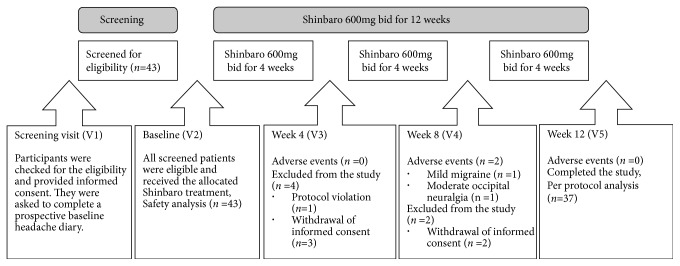
The study flow.

A total of 37 subjects who had completed 12 weeks of Shinbaro treatment were included in the efficacy analyses. This excluded one subject who had been dropped out due to protocol violation in the eligibility criteria and five subjects who had withdrawn their consent for unspecified reasons during the treatment period. Two participants each experienced a minor occipital neuralgia and a moderate migraine attack, which were uncaused by the study medication. Both cases were reported to IRB, and the subjects completed the study participation without further complications. No vital signs or laboratory abnormalities were observed during the study. 81% of the participants were women and 19% were men, and the mean age was 51.5 years. The participants' characteristics are outlined in Table 1, and Figure 1 shows the study flow. 

**Figure 2:**
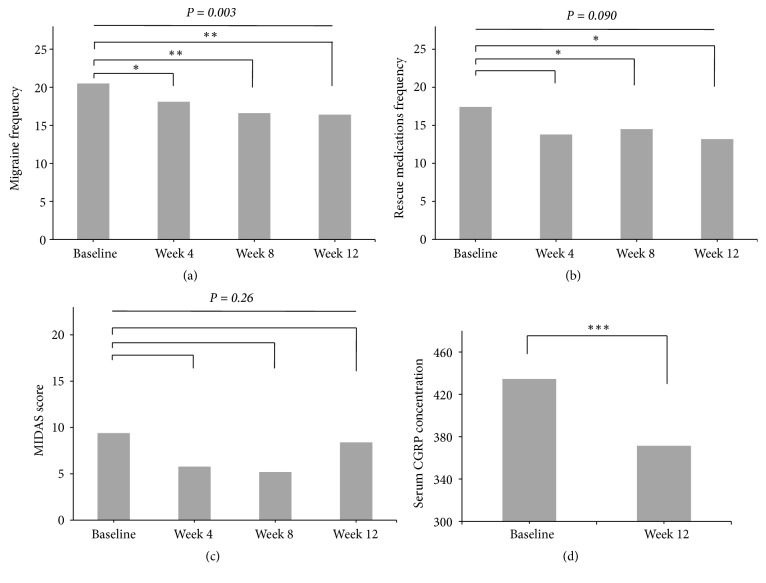
The efficacy variables during 12 weeks of Shinbaro treatment. (a)* Migraine frequency*. (b)* Rescue medications frequency*. (c)* MIDAS* (d)* Serum CGRP concentration measured in pg/mL*.* P*-values in italic were obtained from the one-way repeated measures ANOVA. The migraine frequency decreased time-dependently (*P* =0.003), whereas changes to the rescue medications frequency and the MIDAS score were not time-dependent (*P* > 0.05). The paired t-tests showed that differences of the means were significant at **∗*** P* < 0.05, **∗∗*** P* <0.01, and **∗∗∗*** P* <0.001 significance levels. Per protocol analysis,* n* =37.

The mean migraine frequency of the participants was 20.5 ±7.6 days at baseline and continued to decrease at 4-week intervals down to 16.4 ±9.4 days at week 12 (*P* =0.003) (Figure 2(a)). The overall mean % reduction after 12 weeks of treatment was 19%, and about 22% of the participants showed ≥50% reduction in their frequency after 12 weeks of treatment. There was a significant time effect in that the differences of the means at sequential follow-ups were statistically significant; F (3,108) = 4.85,* P* =0.003 (Figure 2(a)). The LSD t-tests indicated that the differences were significant at week 4 vs. baseline, and also at week 8 vs. baseline (Figure 2(a)). The mean rescue medications frequency was 17.4 ±10.3 days at baseline and 13.2 ±11.6 days at week 12, which was significantly different (*P* =0.035) (Figure 2(b)). Although there was a significant mean difference in the medications frequency by week 8 vs. baseline (*P* =0.024), the* P*-value from the ANOVA indicated that the effect of time was not significant; F (2.43, 87.52) = 2.35,* P* =0.090 (Figure 2(b)). The degree of freedom was adjusted using the Huynh-Feldt estimates of Sphericity (*ε* =0.76). The MIDAS did not decrease significantly at week 12 (8.4 ±17.2) vs. baseline (9.4 ±14.2);* P *=0.79 (Figure 2(c)). No significant time-dependent MIDAS differences could be observed at week 4 nor at week 8 vs. baseline, after correcting for the Greenhouse-Geisser estimates of Sphericity (*ε* =0.52); F (1.57, 56.40) =1.37,* P* =0.26 (Figure 2(c)). The serum CGRP concentration at week 12 was 371.4 ±63.9 pg/mL, which was significantly lower than 434.6 ±59.2 pg/mL of baseline,* P *<0.001 (Figure 2(d)).

## 4. Discussion

The participants' migraine frequency began to decrease significantly within four weeks of the treatment initiation and continued to decrease throughout the treatment period. These fast onset and persistence of therapeutic effects are important attributes of a migraine prophylaxis, considering that patients often fail to adhere to the recommended dose and duration of a treatment when migraine recurs after therapeutic effects shortly subside, or when various adverse events occur even before therapeutic effects are set [25, 26]. For instance, Hepp et al. (2014) reported that adverse events were the main causes for discontinuation among patients who were treated with topiramate (24%) or amitriptyline (17%), whereas other reasons such as patients' choice and loss to follow-up contributed little to the discontinuation rate [27]. In comparison, participants of the current study experienced relatively low rate (5%) of mild and moderate adverse events that were uncaused by the study drug and completed the study without further safety concerns. Furthermore, observing a significant reduction in the acute medications frequency implies that migraine complications such as MOH from the sustained use of acute treatments may be prevented and responses to the medications may be enhanced in a long term.

The central roles of CGRP in the migraine pathophysiology have been well-established ever since increased levels of CGRP were observed from the peripheral blood and saliva of patients who were experiencing migraine attacks [28, 29]. Although there are conflicting findings that CGRP is elevated outside migraine attacks—implying that CGRP is a biomarker for CM, but not so much for EM [30], or that CGRP does not reflect patients' migraine status at all [31]—the general concept that CGRP reflects the trigeminal activation during migraine has been carried forward to pioneer novel migraine therapeutics [32, 33]. Several CGRP receptor antagonists were tested first and showed promising clinical results for the migraine prevention, although some of them had considerable side effects on the liver [34, 35]. More recently, three monoclonal antibodies against CGRP, “ALD-403” (developed by Alder Biopharmaceuticals), “LY2951742” (developed by Arteaus Therapeutics and acquired by Eli Lilly and Co.), and “TEV-48125” (developed by Labrys Biologics-Pfizer and acquired by Teva Pharmaceuticals), and a monoclonal antibody for the CGRP receptors “AMG334” (codeveloped by Amgen, Inc. and Novartis) were found to have the same antimigraine effects but with more prolonging effects—they are injected monthly—and better safety profiles and target specificity [35, 36]. As of September 28, 2018, “LY2951742” or also called “Galcanezumab-gnim” received the United States FDA approval as a preventive treatment of migraine.

Even though clinical results from the recent studies of CGRP antibody therapeutics are promising, we suggest that Shinbaro may have several advantages over the direct CGRP binding proteins. First of all, the orally administered Shinbaro can prevent common adverse events that are caused by the injection procedure such as injection-site pain and discomfort and, therefore, yield higher adherence and compliance from the patients [36]. Next, because CGRP is involved in multiple physiological functions, direct blockade of CGRP or its receptors may cause considerable long-term safety issues. For example, CGRP is a potent vasodilator that may also protect against several cardiovascular diseases such as cardiac ischemia and vasospasm [37]. Moreover, the long-term efficacy and safety of the CGRP antibodies still need to be established through anti-drug antibodies testing to prevent immunogenicity problems, and immune tolerance to the drug at low dose does not give a complete safety assurance [37, 38]. On the other hand, Shinbaro has been prescribed to patients with OA or inflammatory disorders for almost a decade now, and its herbal components give the drug a better safety profile. Lastly, the initial market prices for the CGRP antibodies can further limit patients' choice, whereas Shinbaro is a cost-efficient medication that can be easily prescribed to patients who visit clinics. Overall, we suggest that Shinbaro, a natural medication of six herbal constituents, is a potent migraine prophylaxis that can be considered a great alternative to the direct CGRP inhibiting molecules that are in trials.

The pharmacological actions of Shinbaro have been well-established through previous studies in OA rats and patients. In 2016, HK Cho et al. examined the dorsal root ganglion tissues of the lumbar disc herniation rats and found that the group that was treated with 300mg of Shinbaro showed downregulated neuroglial activity, as well as decreased expressions of CGRP and transient receptor potential cation channel subfamily V member 1 (TRPV1), compared to the aceclofenac treated group or to a vehicle control group [14]. Importantly, attenuation of the pain behaviors was observed through the course similar to that of CGRP and TRPV1 downregulation, and the reduction of TRPV1, an integrator of multiple sensory inputs, may particularly explain the antinociceptive mechanism of Shinbaro. Among the six constituents,* Cibotii *Rhizoma and* Eucommiae *Cortex demonstrated strong analgesic effects, whereas* Cibotii *Rhizoma,* Achyranthis *Radix,* Eucommiae *Cortex, and* Ledebouriellae* Radix were reported to have strong anti-inflammatory effects in various inflammatory pain models [16, 17]. Moreover, it has been already established that these herbal constituents can inhibit nitric oxide production and reduce the TNF-*α* and COX-2 serum and protein levels [39–41]. Modulation of such proinflammatory mediators and cytokines is an effective anti-inflammatory mechanism that can contribute to the downregulation and inhibition of CGRP, which is the central player of the migraine pathophysiology [9–13]. The current study's finding that Shinbaro administration reduced the serum CGRP level is consistent with the findings from the earlier OA studies and supports the proposed pharmacological actions of Shinbaro, also in migraine.

However, this study results should be taken as preliminary data, and further studies should corroborate that Shinbaro can efficiently decrease the blood CGRP level and, therefore, treat migraine. First, we suggest that the correlation between the migraine frequency reduction and the serum CGRP reduction must be established. That is, although only the serum CGRP samples at baseline and week 12 were collected and compared according to the study protocol, additional blood collections and CGRP analysis at week 4 and week 8 would demonstrate that the migraine frequency was reduced in relation to the serum CGRP reduction along the administration period. Additionally, several factors that can affect the CGRP level at the time of blood collection, such as patients' migraine status, duration of the headache or migraine attacks, and the time away from rescue medications intake, should be taken into account to obtain more reliable measures [29–31]. Furthermore, because this is a single-arm, nonrandomized study, the placebo effect and subject bias cannot be eliminated. To address these issues, participants should be assigned to different dose groups or to a control group in future double-blinded, randomized, controlled studies. In addition, generalization of our findings regardless of patients' migraine types can be misleading. For example, Wang et al. (2013) pointed out that EM and CM patients have significantly different migraine features, symptoms, psychological states, and impacts on daily lives [42]. Distinctions were further made for CM with MOH patients from those with migraine without aura [43]. In light of this literature, the fact that Shinbaro did not improve MIDAS of the participants may be explained by the variation in the migraine types among the participants [44]. Therefore, further classification of the migraines, based on the internationally accepted standardized migraine criteria, and subgroup analyses may be necessary with increased sample size per group. Lastly, because patients of different migraine types can have different responses to Shinbaro, more accurate and possibly higher response rate may be derived by specifying the subgroup population; 22% of the participants showed ≥ 50% reduction in their migraine frequency after 12 weeks of Shinbaro treatment. Proceeding with current study's findings, future studies with improved study design and more sensitive CGRP assay would help to extend therapeutic application of Shinbaro as a prophylactic treatment for migraine.

## 5. Conclusion

Shinbaro, an herbal medication for several bone disorders and inflammatory diseases, was investigated as a migraine prophylaxis in this pilot study. After 12 weeks of Shinbaro administration, the mean migraine frequency, rescue medications frequency, and the serum CGRP concentration decreased significantly compared with baseline. The anti-inflammatory and antinociceptive properties of the herbal components of Shinbaro seem to intervene in the CGRP mediated pathophysiology, thus reducing the migraine frequency. Although further randomized controlled studies with increased sample sizes and more sensitive CGRP assay are still needed, we anticipate that the therapeutic application of Shinbaro may be extended to the treatment of migraine.

## Figures and Tables

**Table 1:** Characteristics of the participants.

Number of participants enrolled	43
Excluded from the study (%)	6 (14%)
Completed the study (%)	37 (86%)
Mean age ± SD^a^	51.5 ±11.6
Sex^a^	
Male (%)	7 (19%)
Female (%)	30 (81%)
Adverse events (%)^b^	2 (5%)
Minor (%)	1 (2%)
Moderate (%)	1 (2%)
Severe (%)	0 (0%)

^a^ Per protocol analysis, *n *=37. ^b^ Safety analysis, *n* =43.

## Data Availability

The .xlsx data used to support the findings of this study are available from the corresponding author upon request.
